# Supplementary figures and images for: Benefits of Telemonitoring of Pulmonary Function—3-Month Follow-Up of Home Electronic Spirometry in Patients with Duchenne Muscular Dystrophy
Source: J Clin Med. 2022 Feb 6;11(3):856. doi: 10.3390/jcm11030856 (PMC8837102; doi:10.3390/jcm11030856)

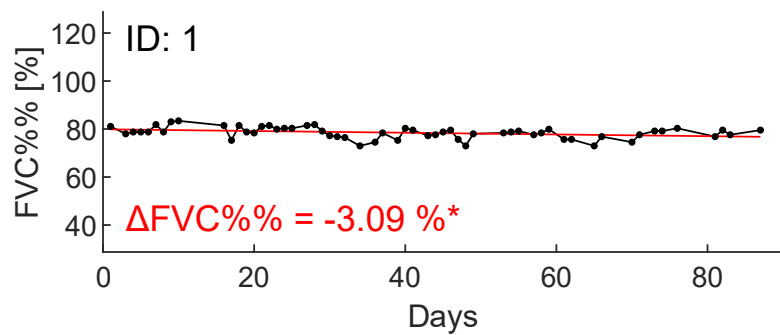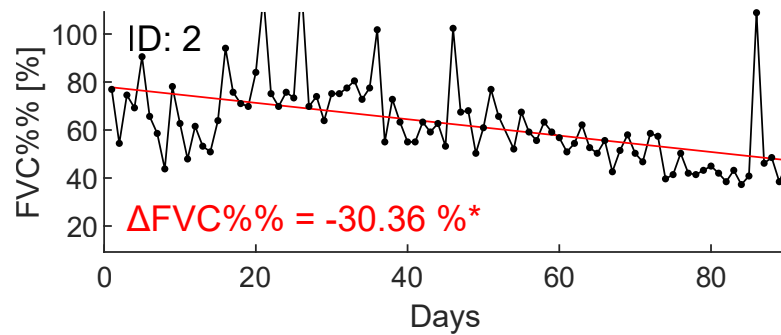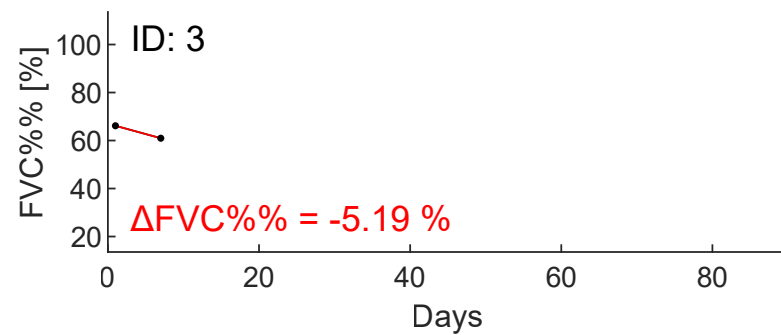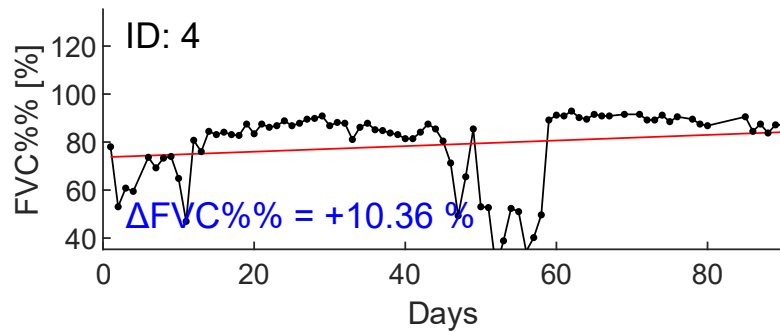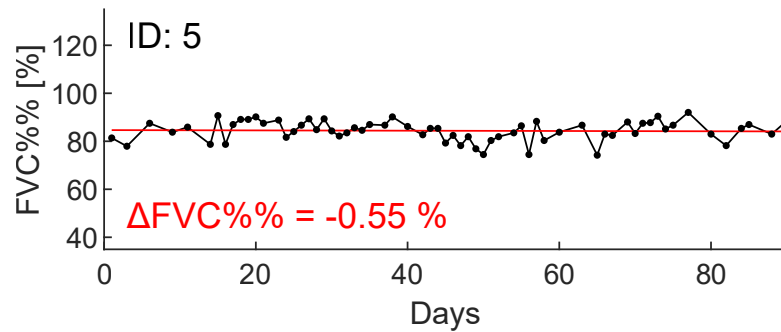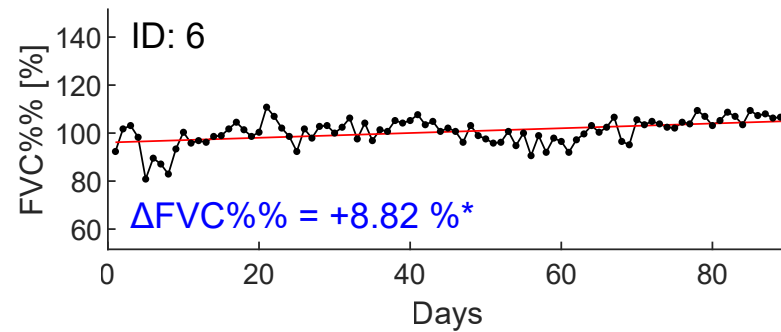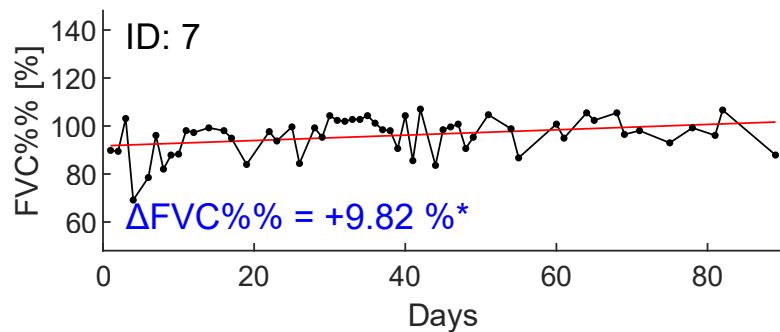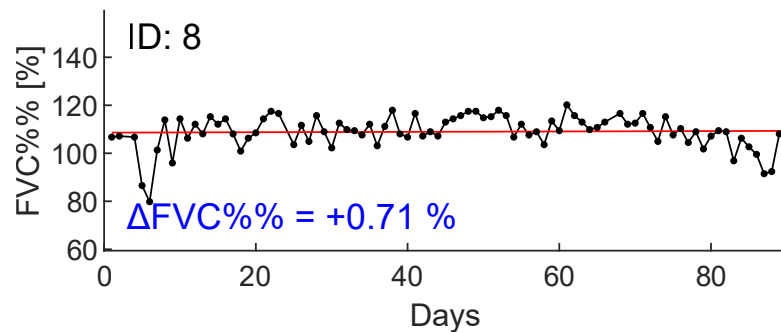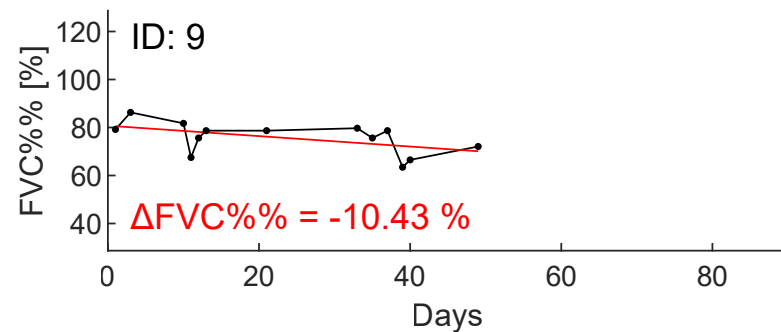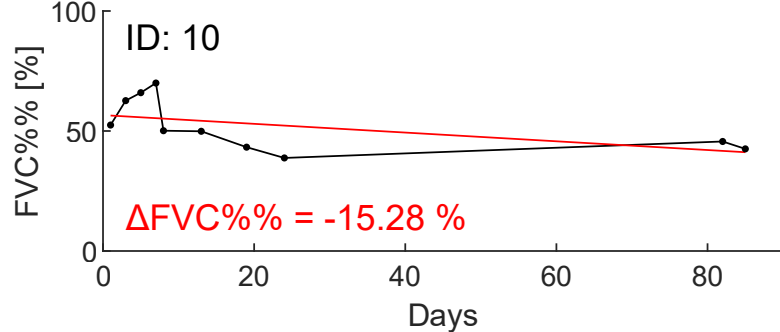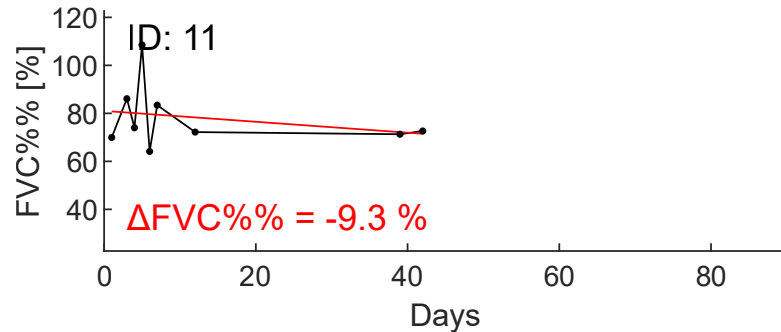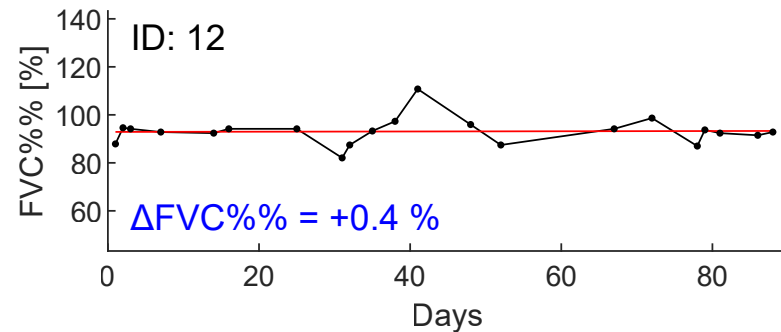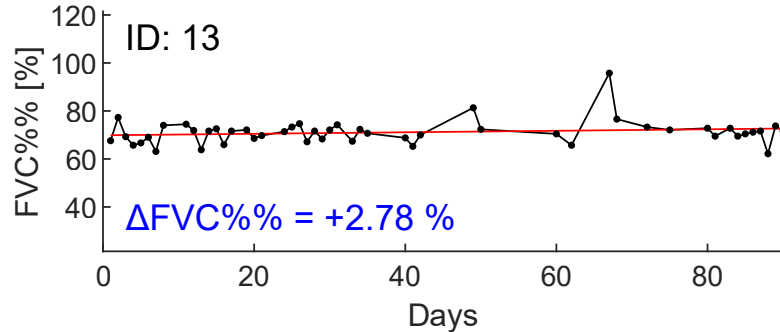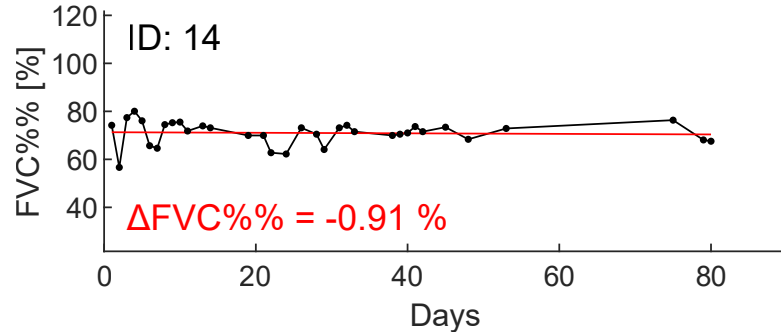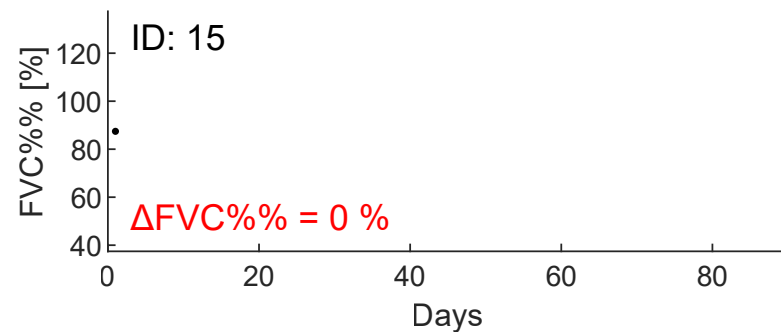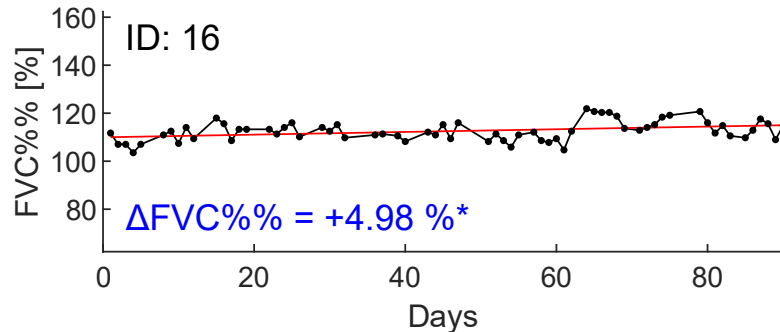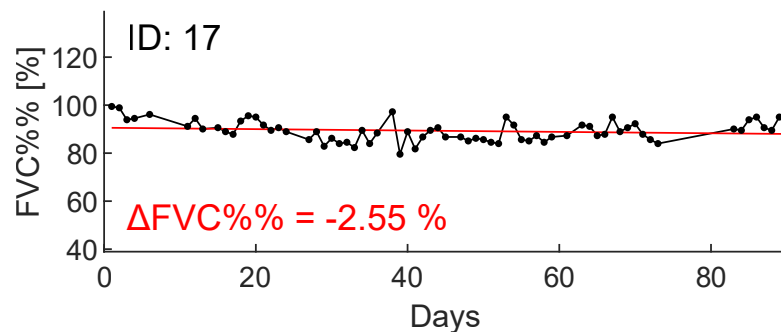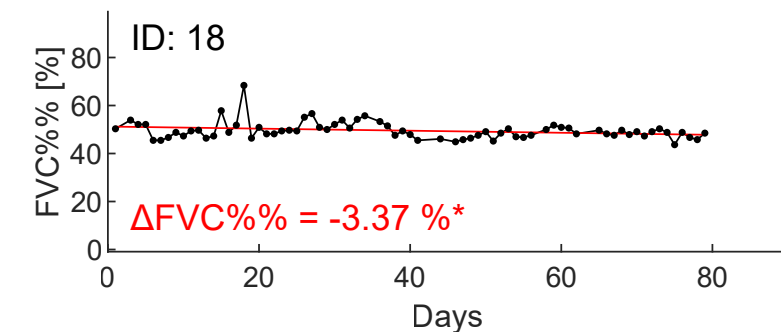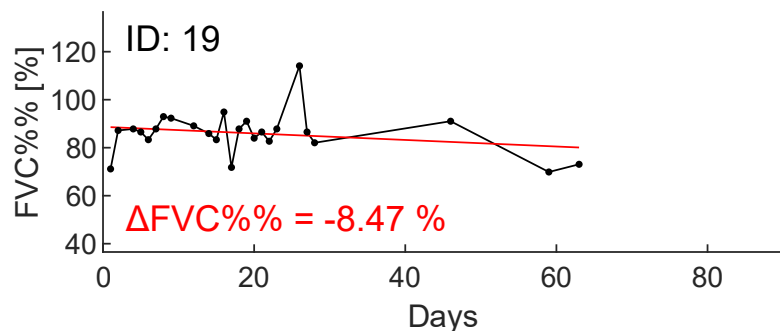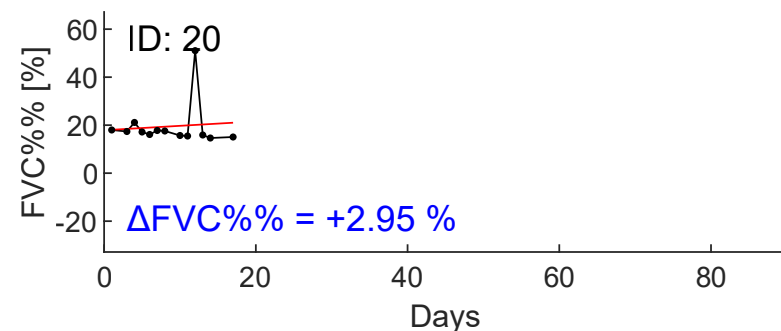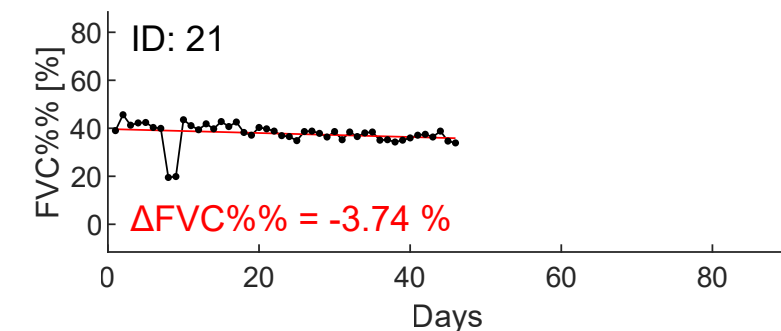

Supplement: Supplementary file 1 [file jcm-11-00856-s001.zip › FigureS1.pdf]

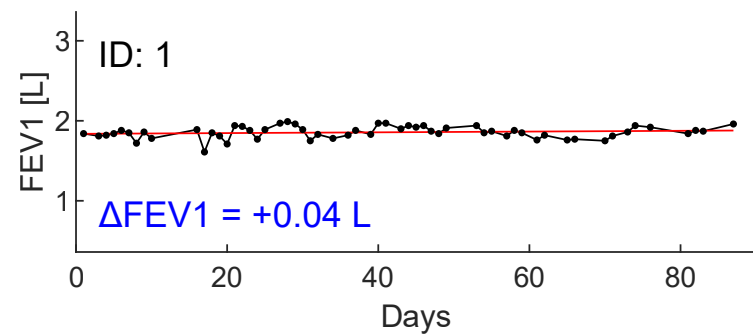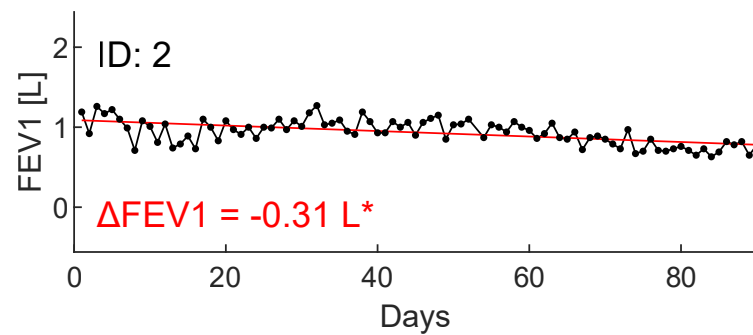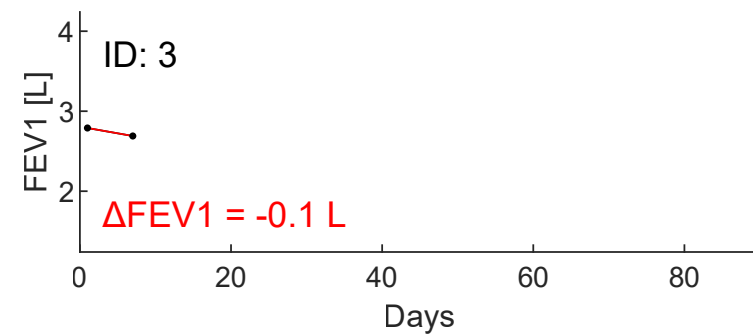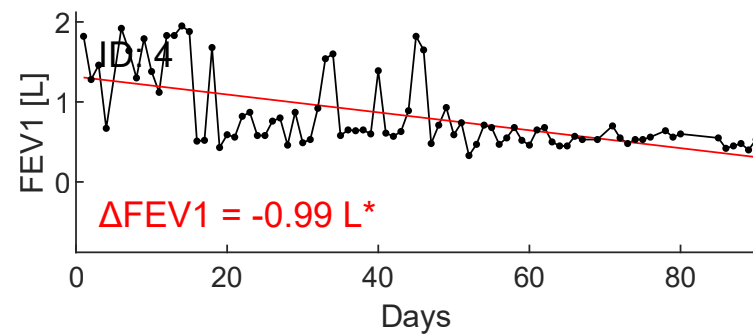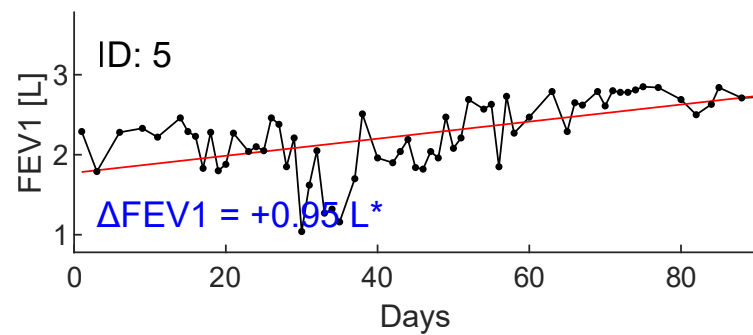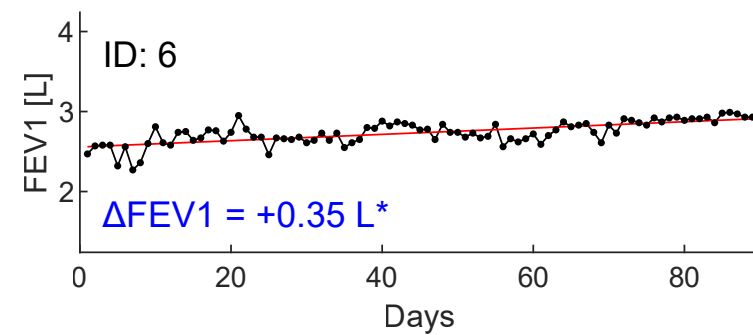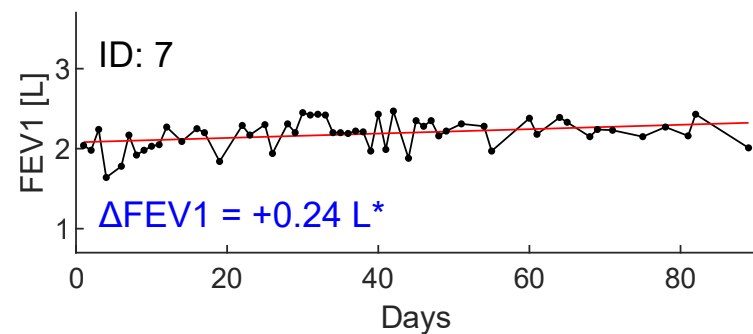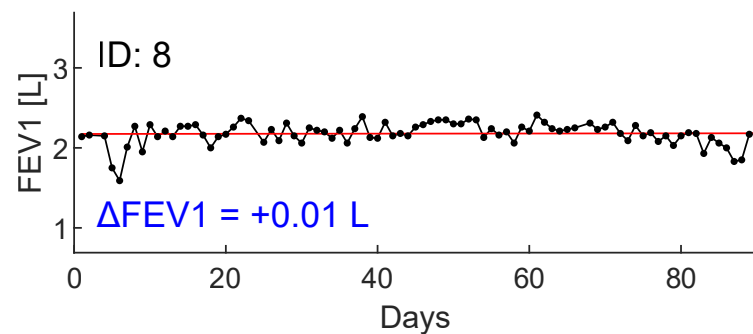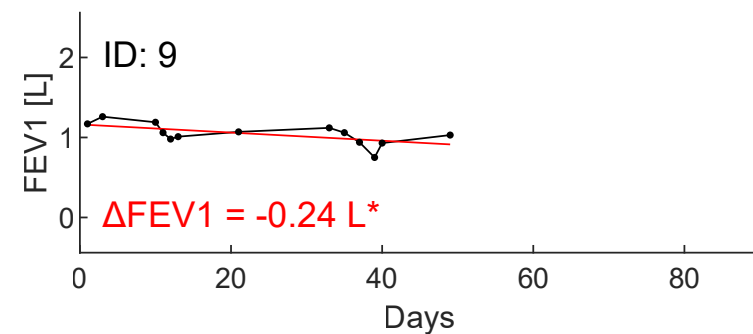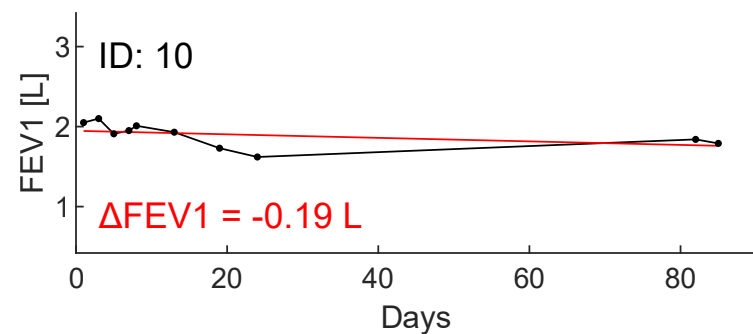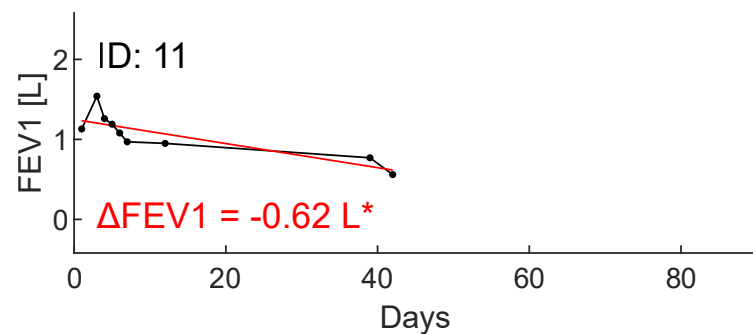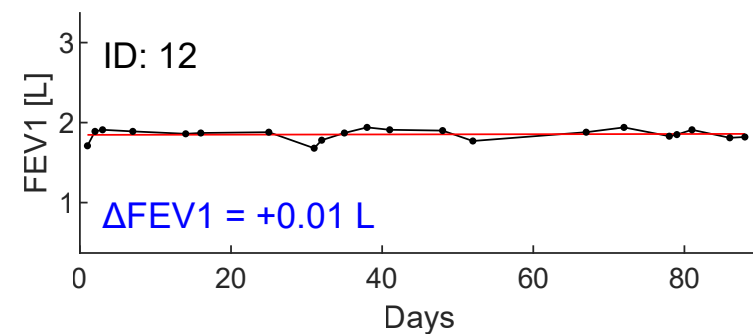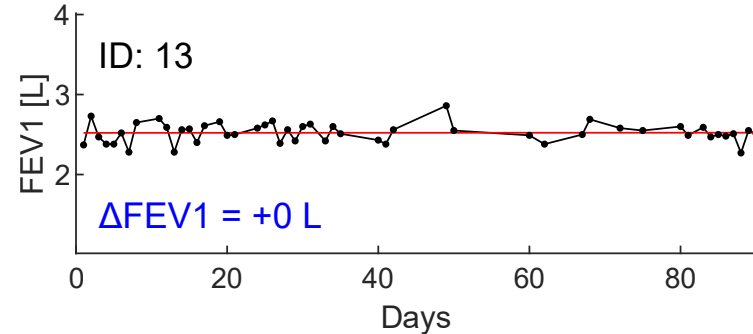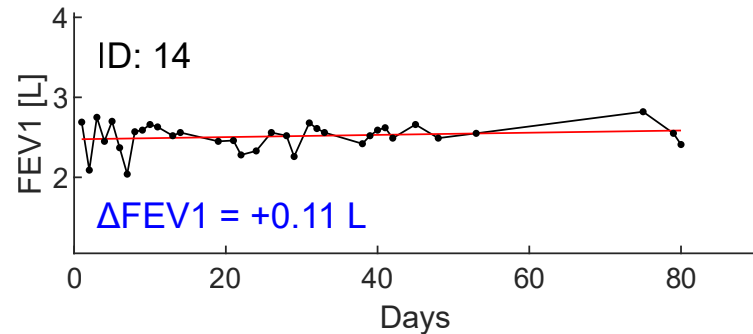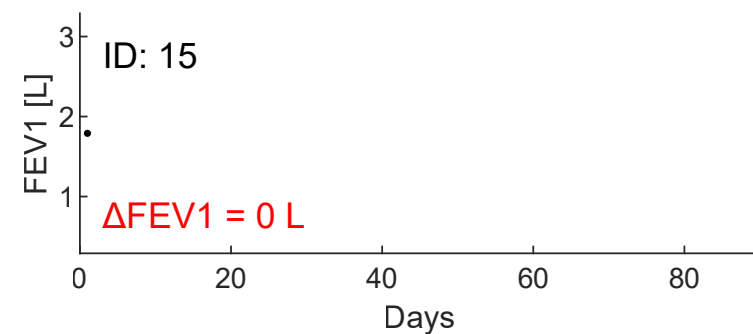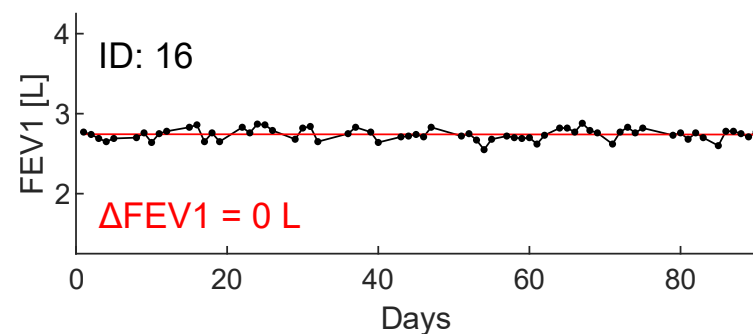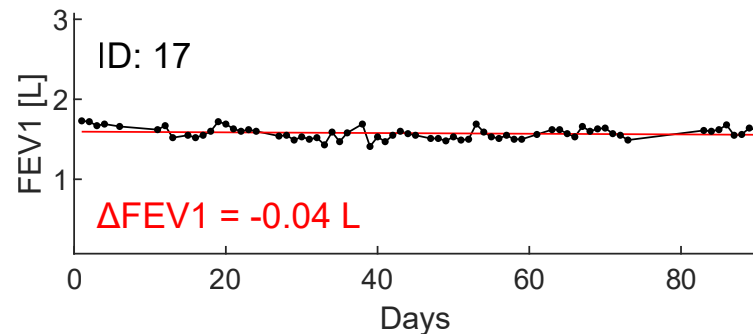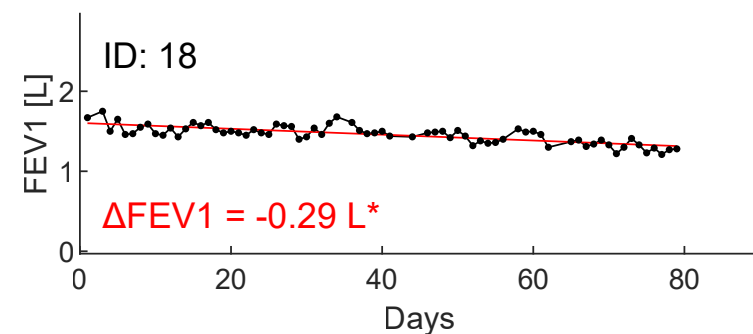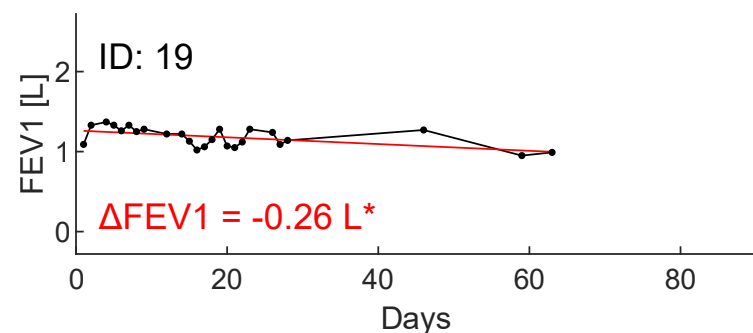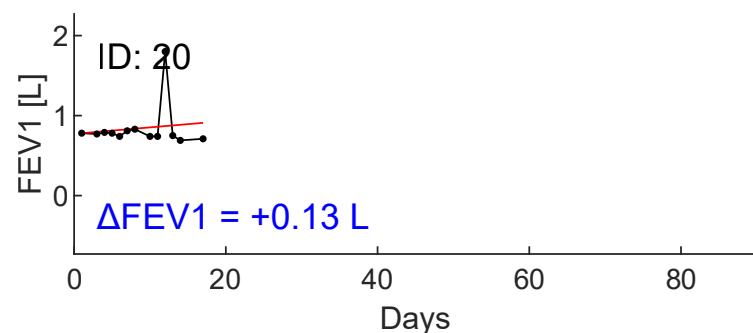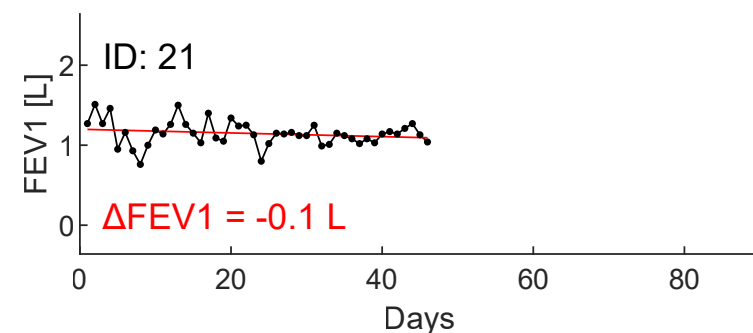

Supplement: Supplementary file 1 [file jcm-11-00856-s001.zip › FigureS2.pdf]

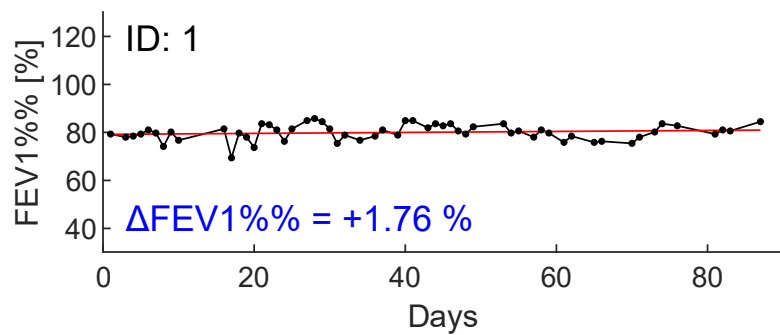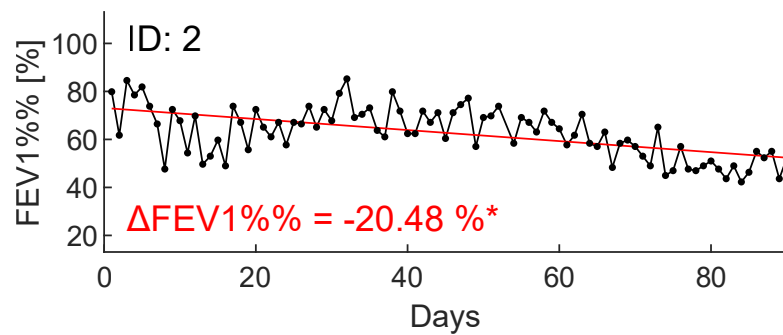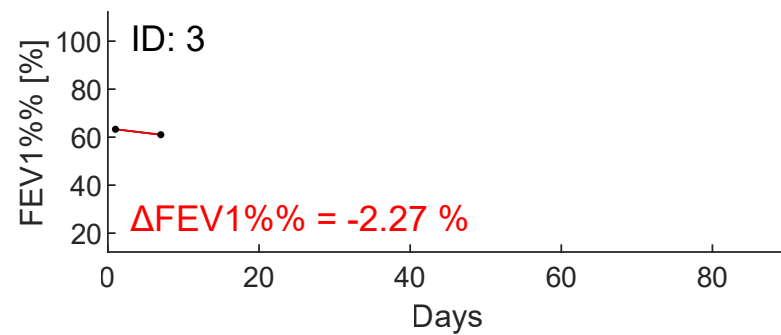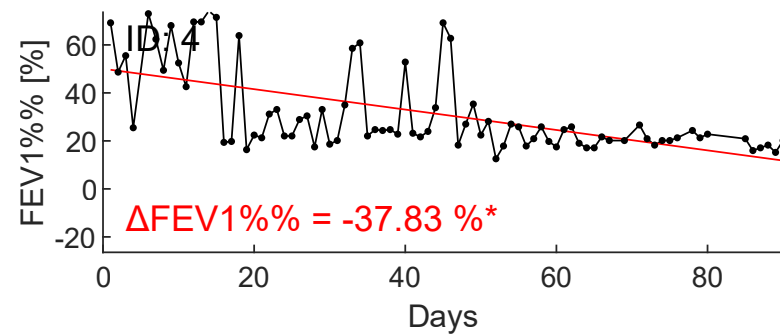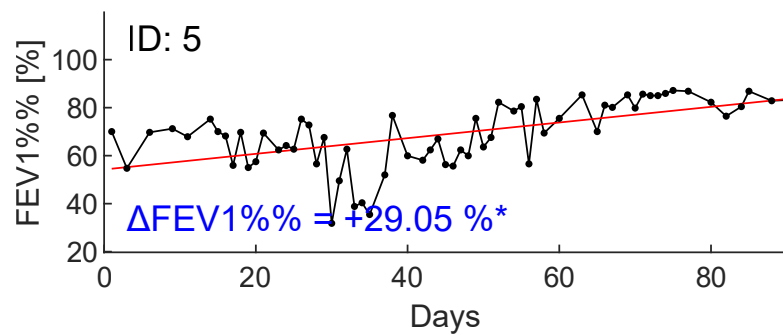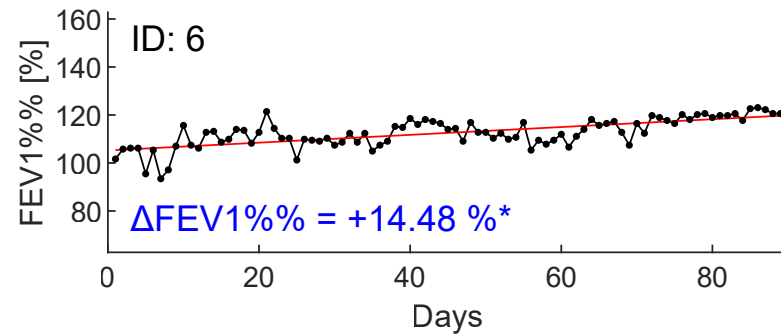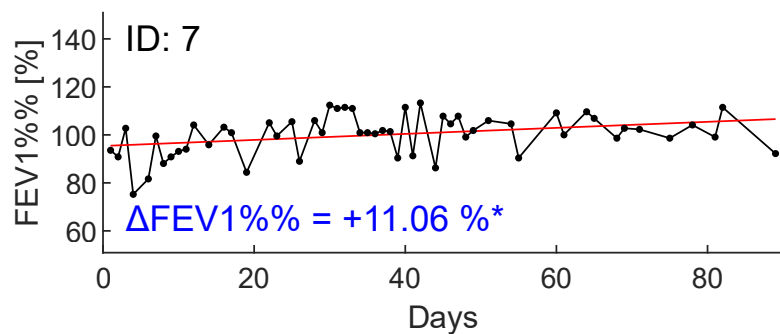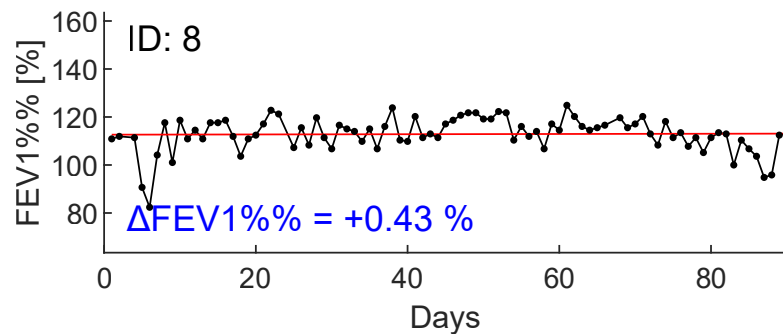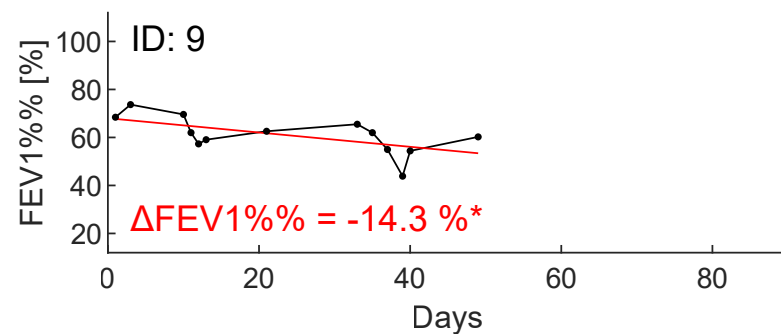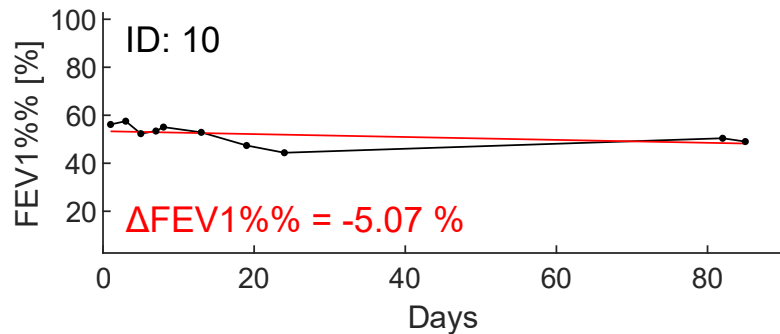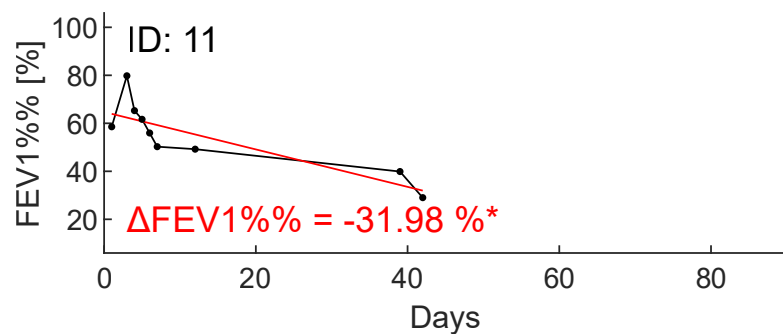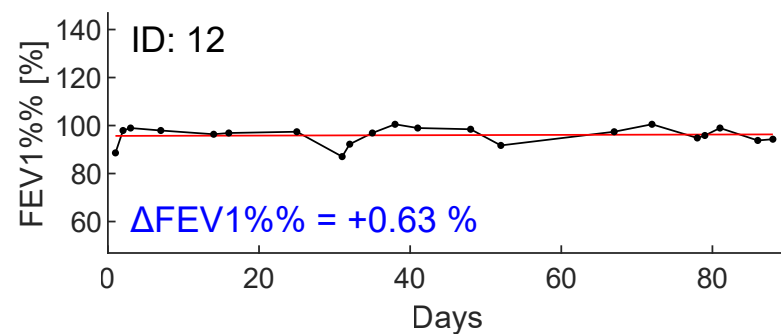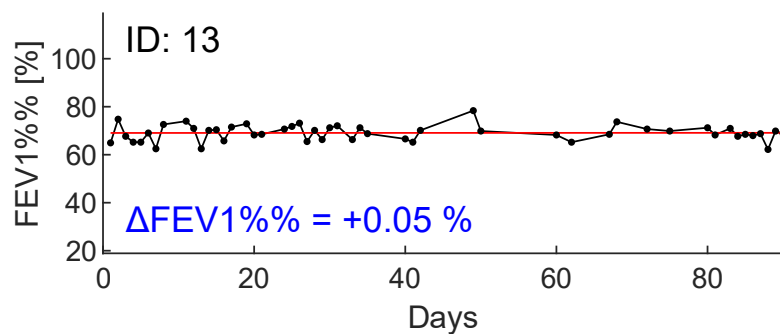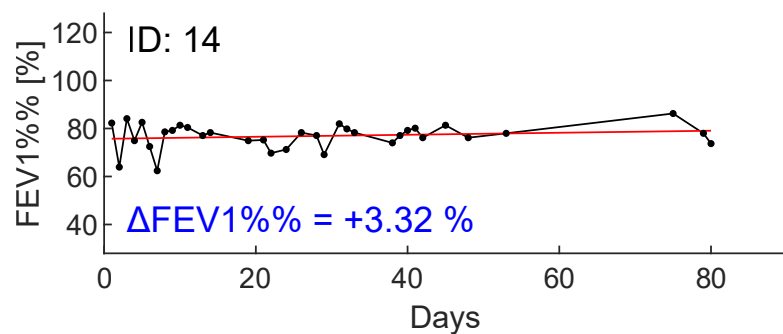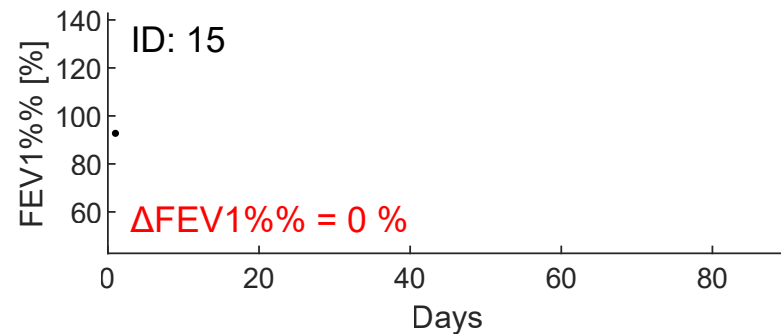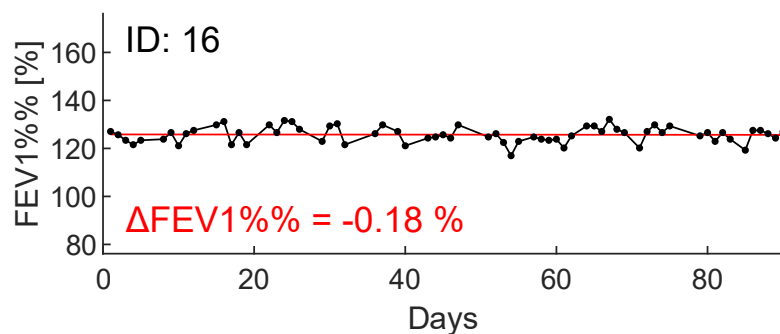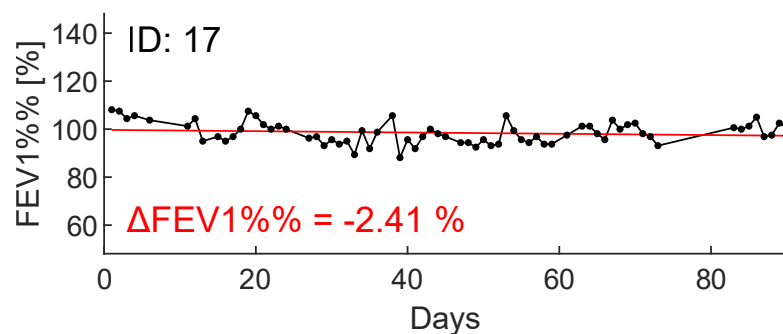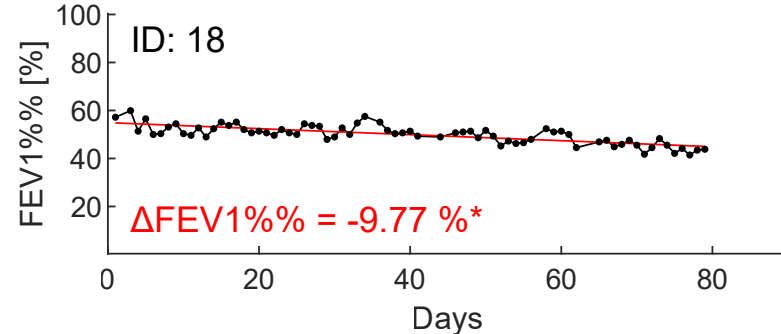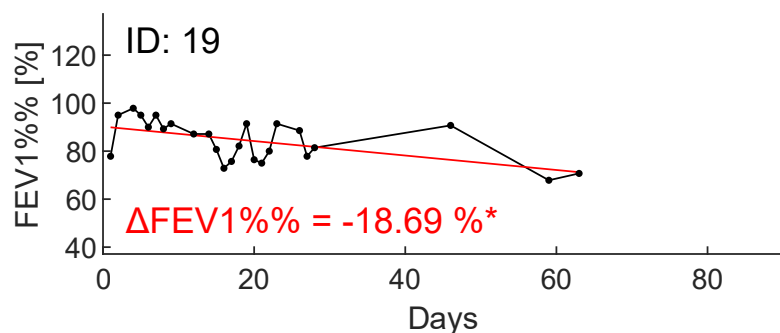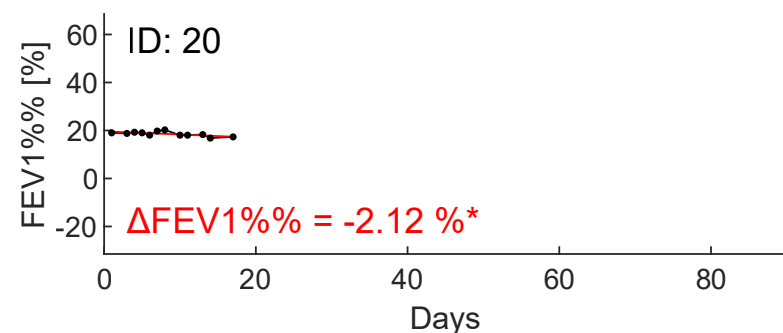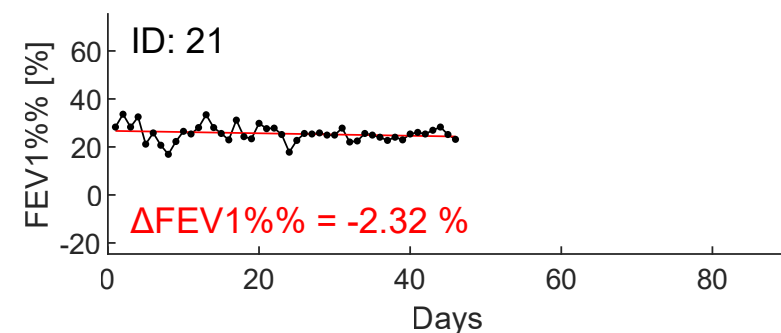

Supplement: Supplementary file 1 [file jcm-11-00856-s001.zip › FigureS3.pdf]

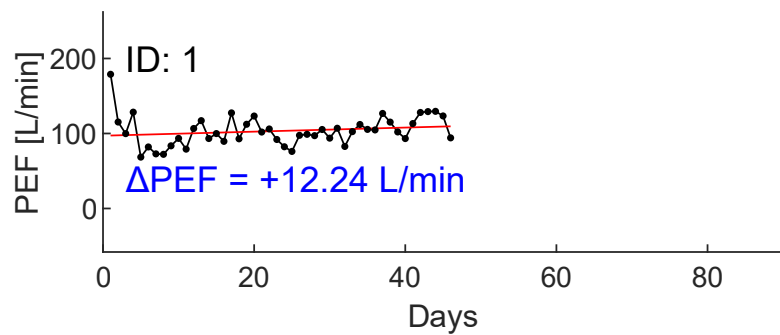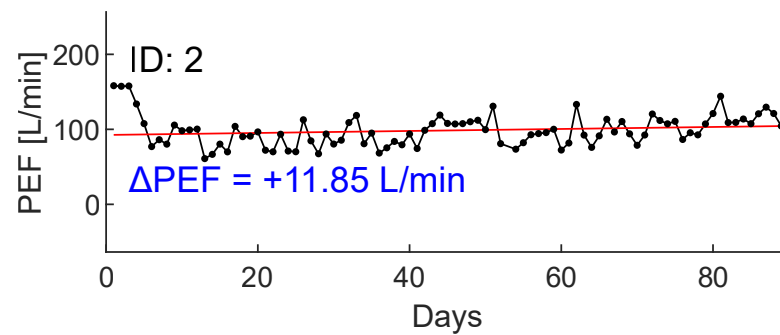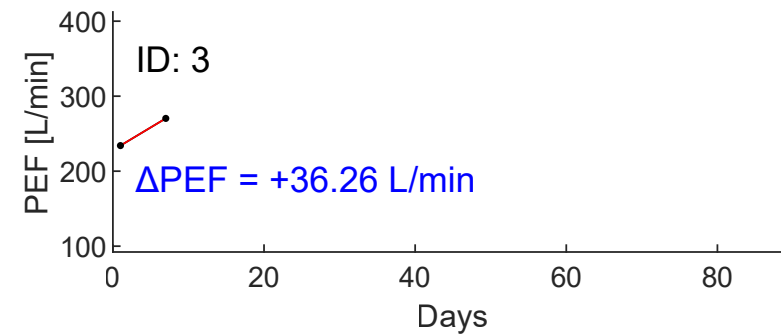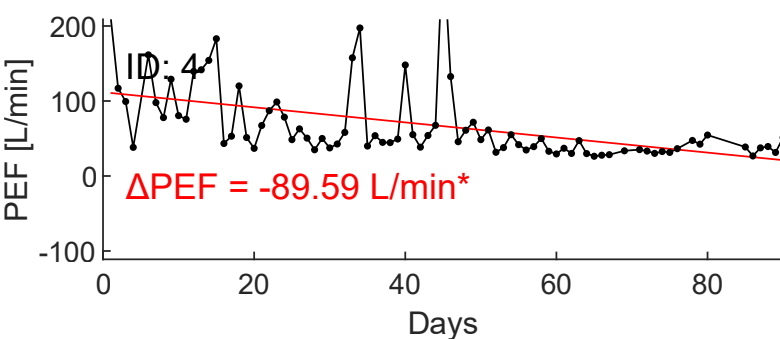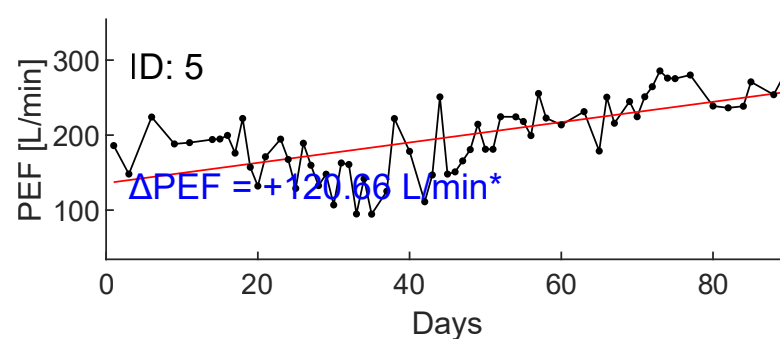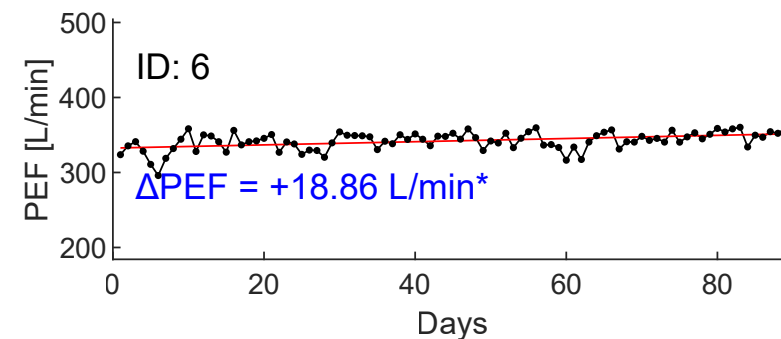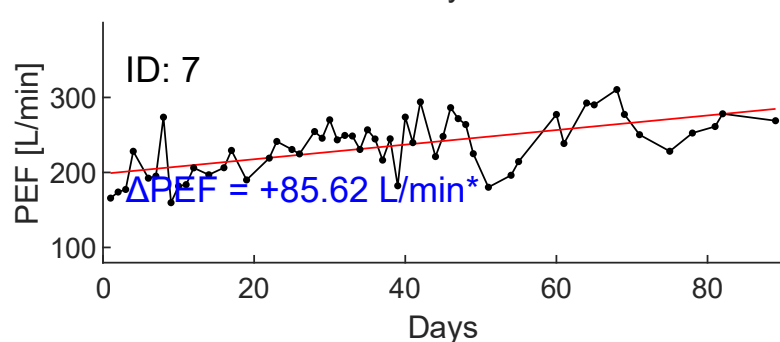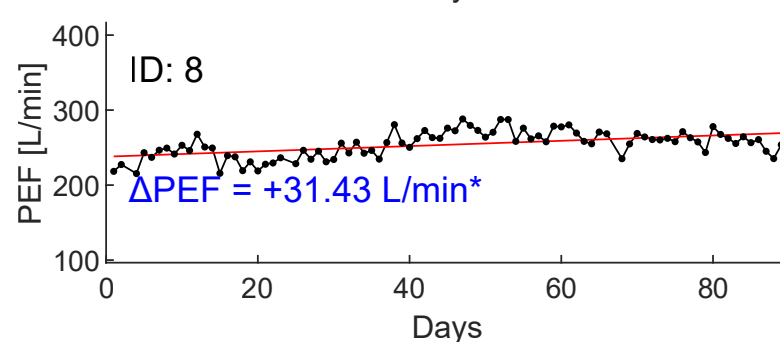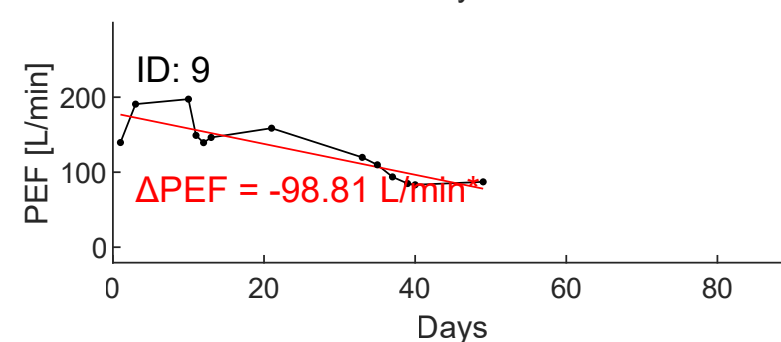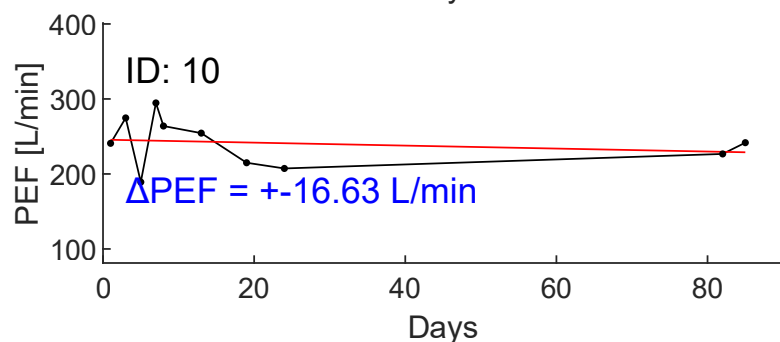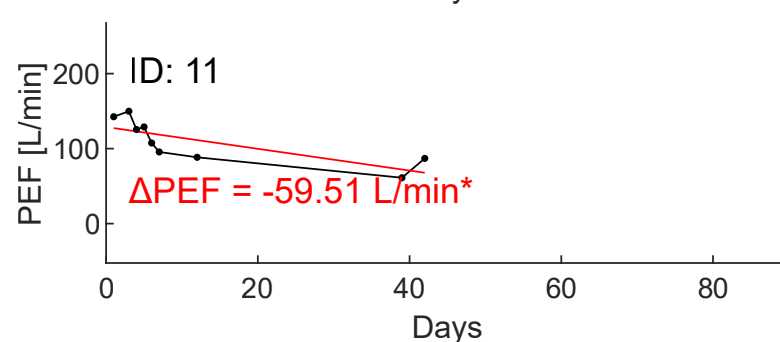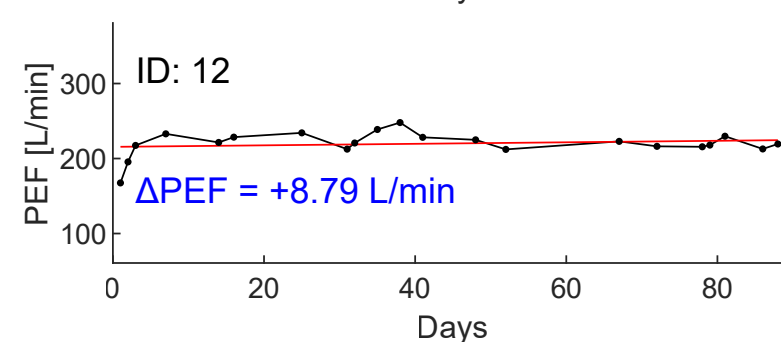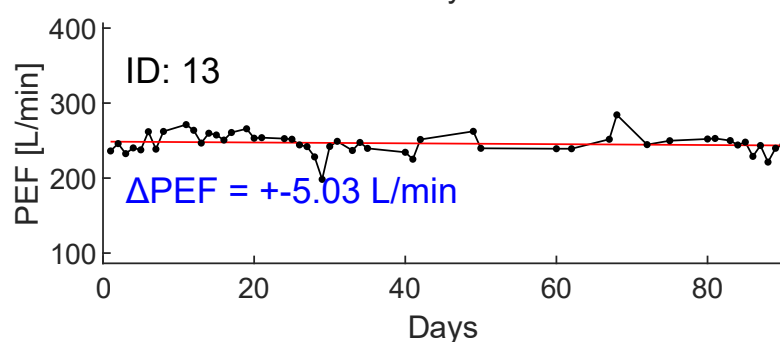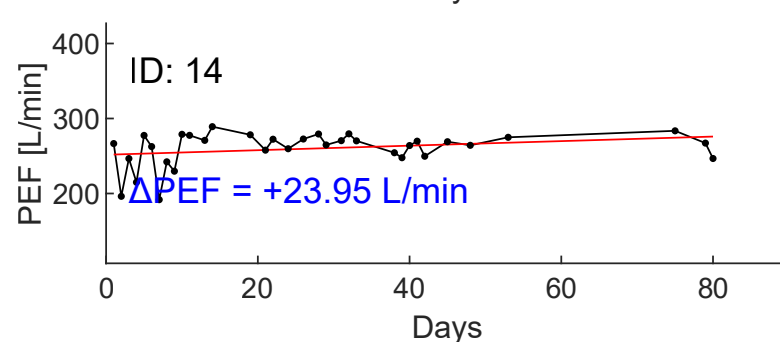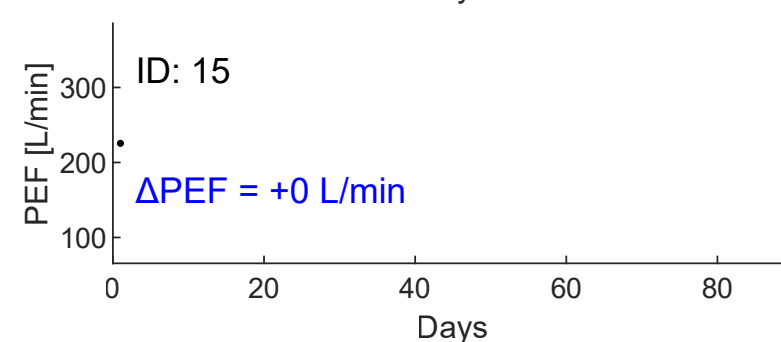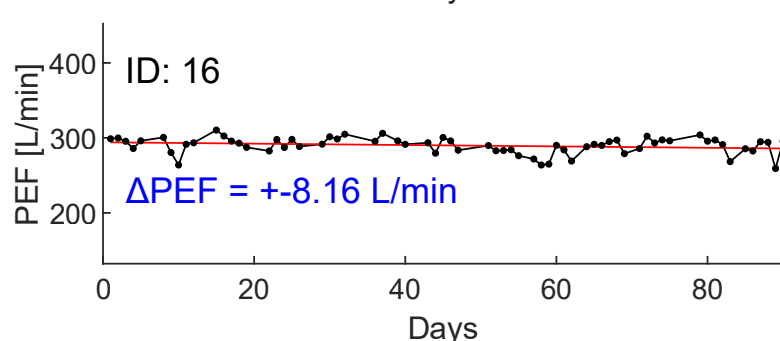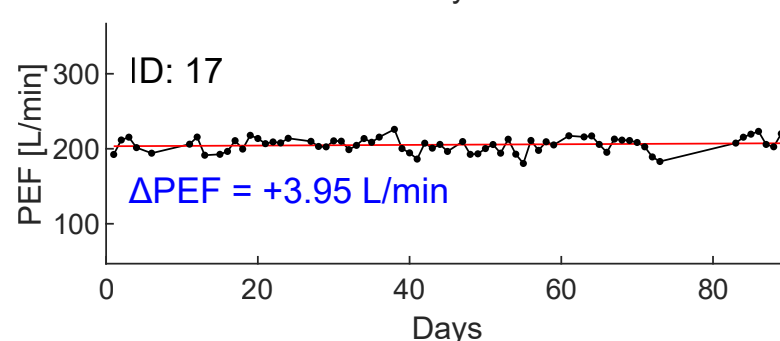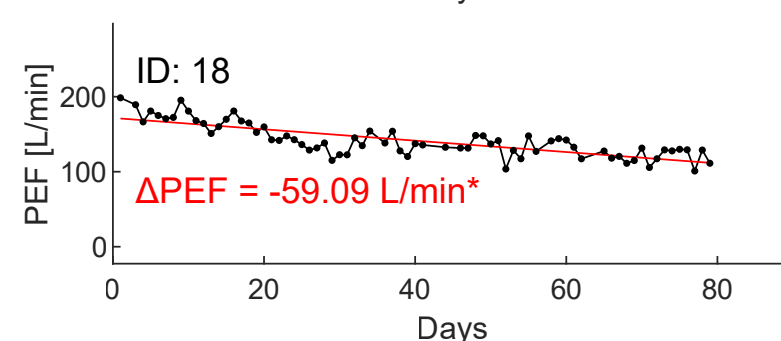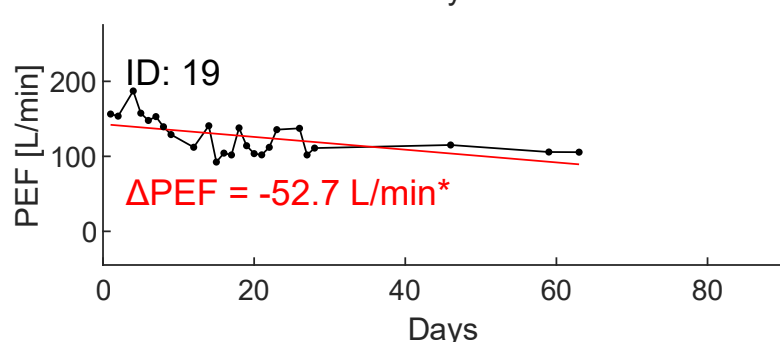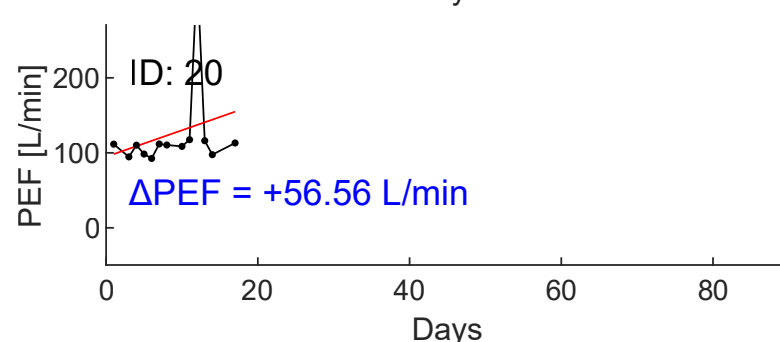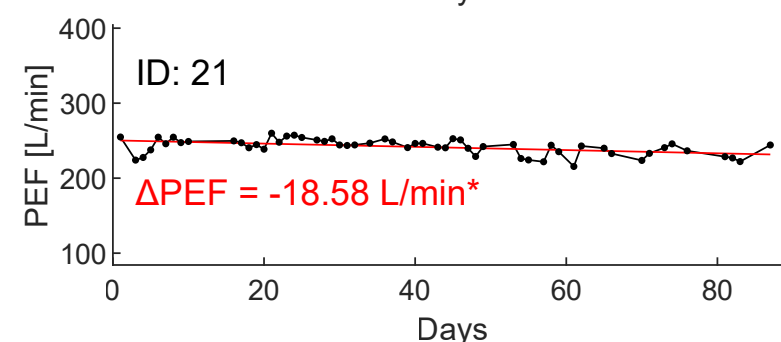

Supplement: Supplementary file 1 [file jcm-11-00856-s001.zip › FigureS4.pdf]

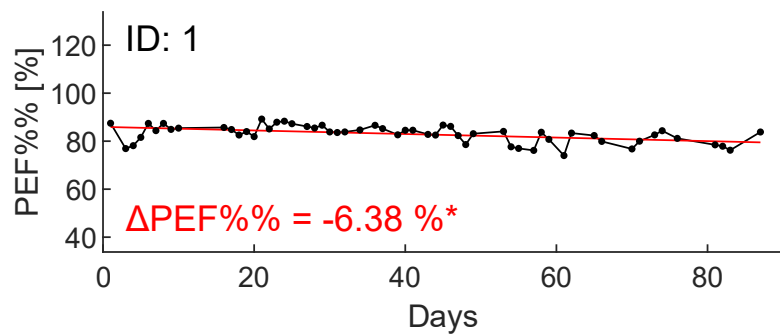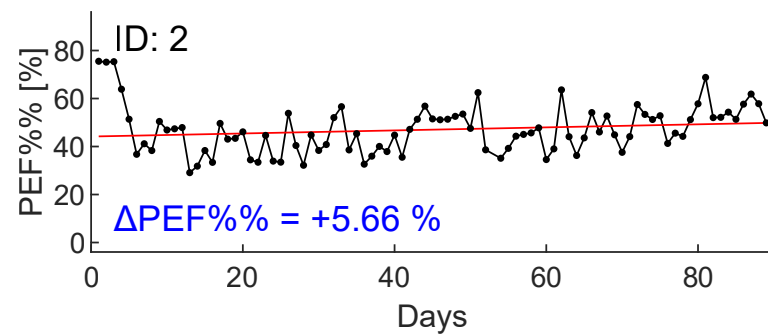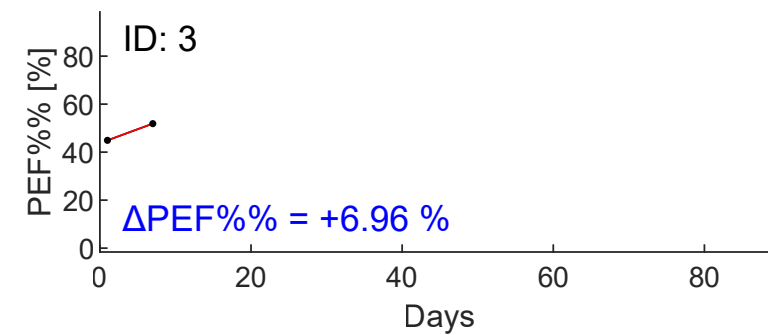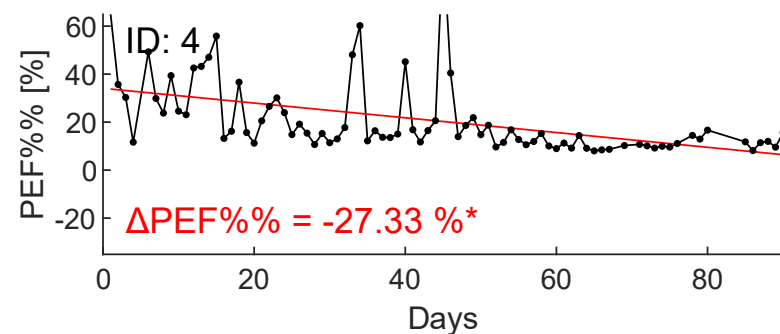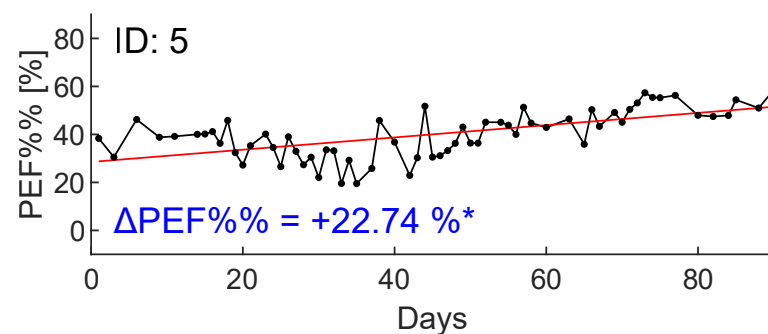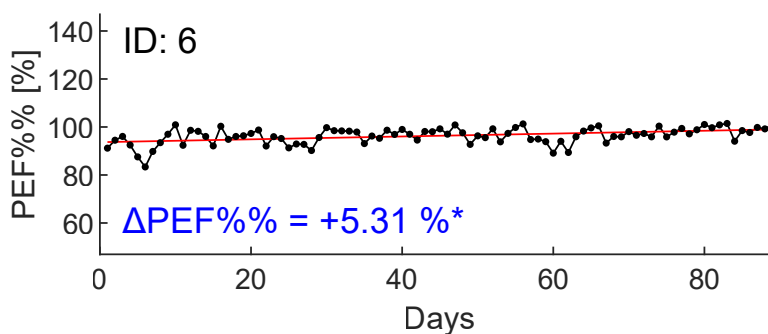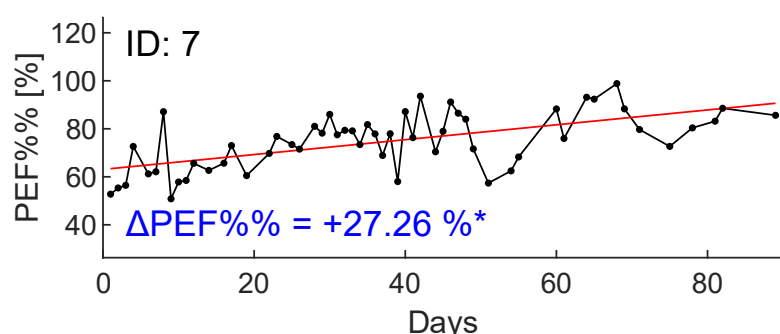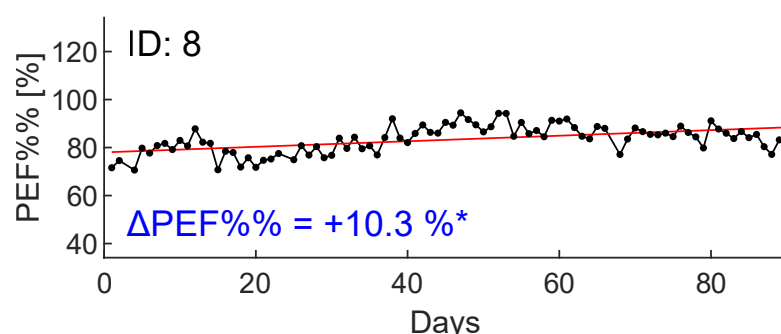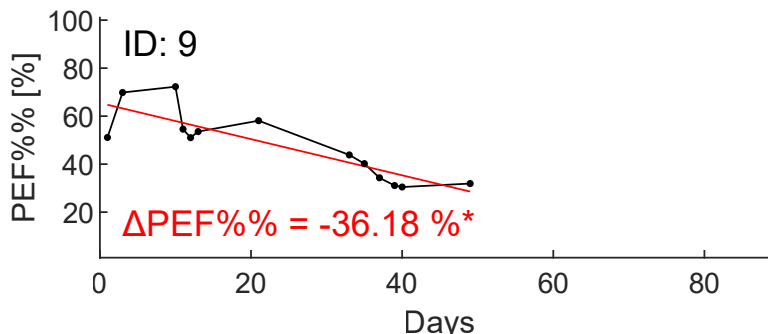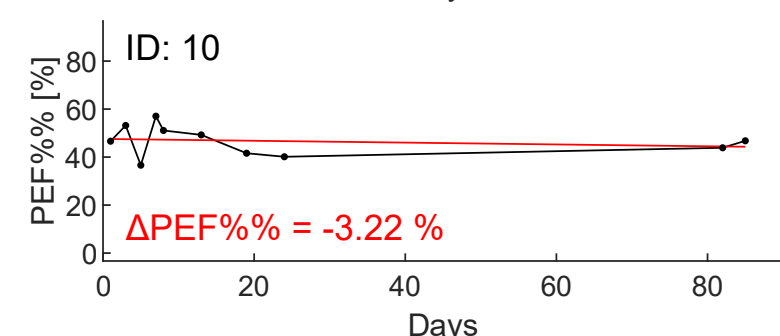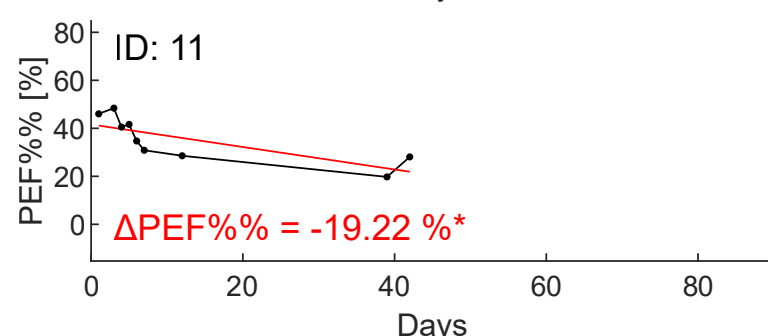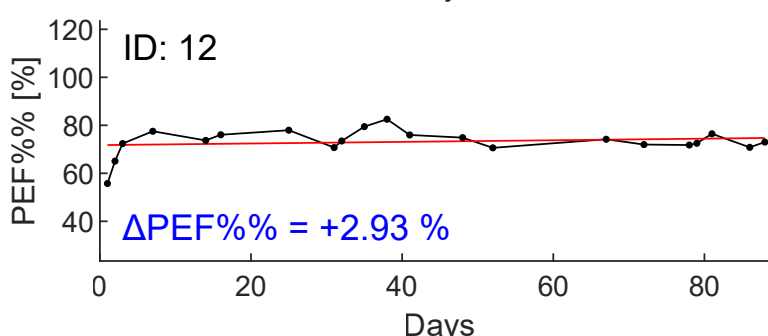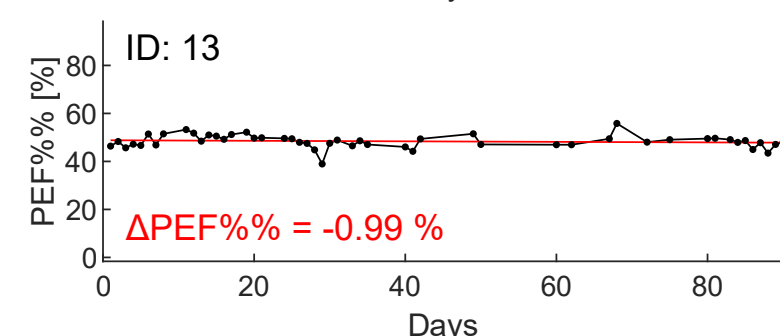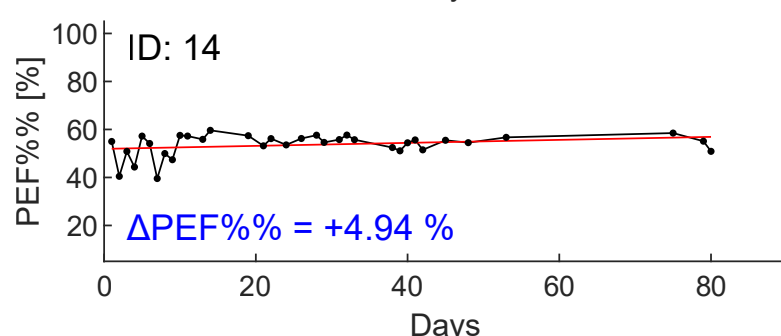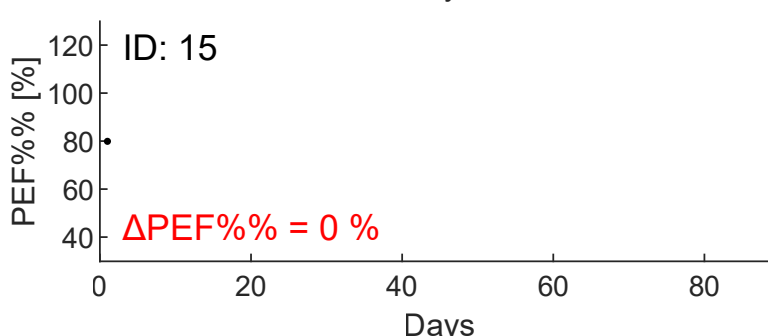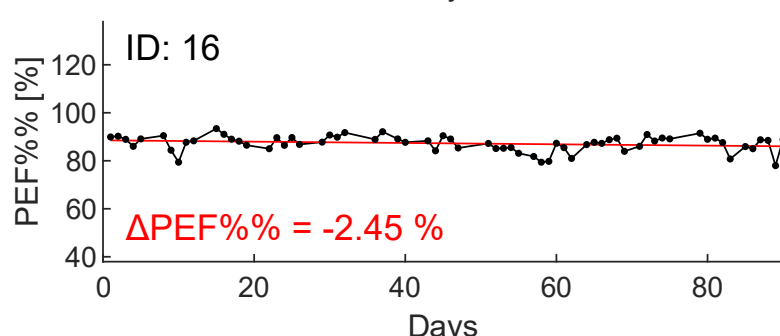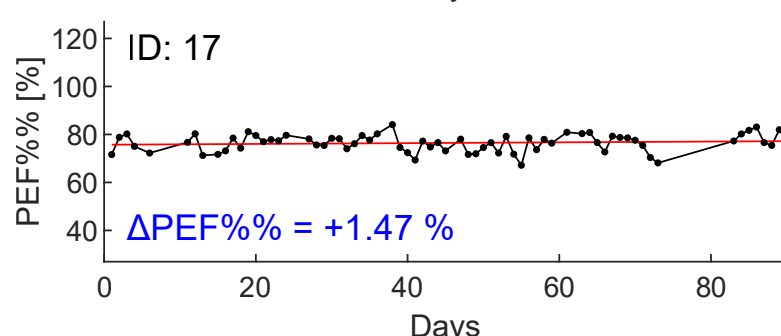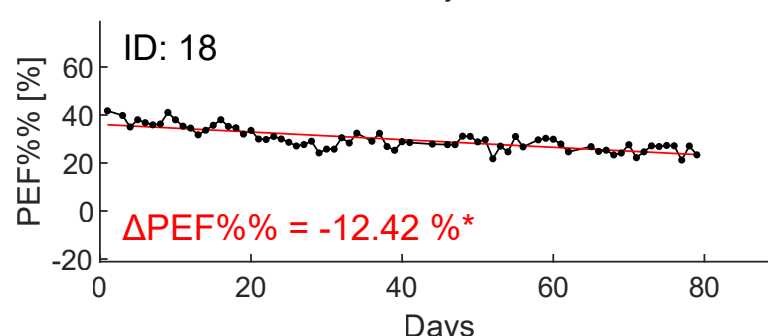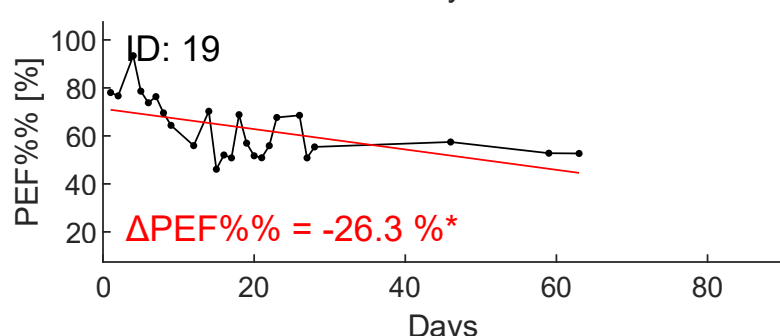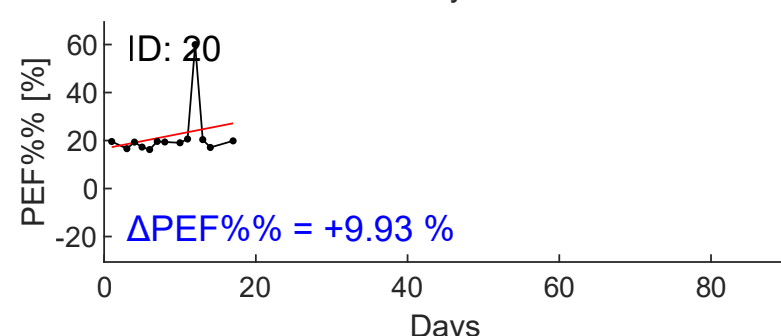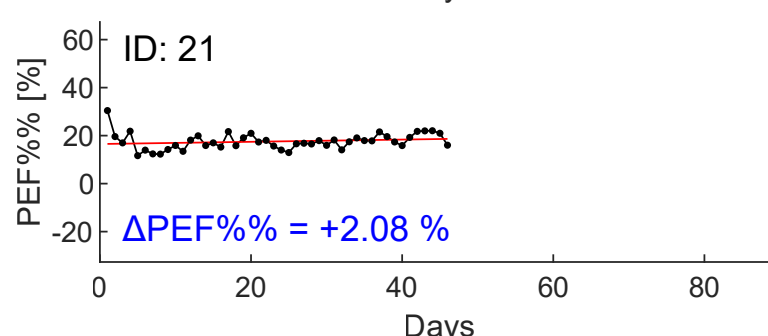

Supplement: Supplementary file 1 [file jcm-11-00856-s001.zip › FigureS5.pdf]
